# 

**Published:** 1891-11

**Authors:** 


### CONTENTS—NOVEMBER, 1891.—VOL. VII, No. 5.

ORIGINAL COMMUNICATIONS—

Some Observations upon the Experimental Use of the Aided Intestinal Suture. Geo. H. Lee, M. D. ....... 157

Hysteria. Fannie Leake, M. D. ....... 163

Discussion on Hysteria ....... 168

The Microscope in Medicine. B. F. Church, M. D. ....... 169

Cancer; What is it? Causes and Treatment. C. S. Reeves, M. D. ....... 173

Removal of the Secundines; The Capiat. J. S. Poynor, M. D. ....... 175

CORRESPONDENCE—

The Pan-American Medical Congress ....... 181

A Novel Treatment for Phthisis Proposed. Letter from Dr. T. J. Turpin ....... 182

More Quackery. Letter from Dr. Fox ....... 183

SOCIETY NOTES—

Northwest Texas Medical Society; Austin District Medical Society, Seventeenth Quarterly Meeting. Programme ....... 184

EDITORIAL—

Alcohol not a Stimulant ....... 187

Austin District Medical Society ....... 189

A Bitter Pill ....... 186

The Capiat ....... 190

Mississippi Medical Monthly ....... 190

Dr. Lee's Article ....... 190

MEDICAL NEWS AND MISCELLANY ....... 191

BOOK NOTICES ....... 194

PUBLISHER'S NOTES ....... 197

